# Supplementary material for: MATE1 Deficiency Exacerbates Dofetilide-Induced Proarrhythmia
Source: Int J Mol Sci. 2022 Aug 3;23(15):8607. doi: 10.3390/ijms23158607 (PMC9369325; doi:10.3390/ijms23158607)
Supplement: Supplementary file 1 [file ijms-23-08607-s001.zip › ijms-1844566-supplementary.pdf]

Supplemental Materials

MATE1 Deficiency Exacerbates Dofetilide-Induced Proarrhythmia

Muhammad Erfan Uddin [1], Eric D. Eisenmann [1], Yang Li [1], Kevin M. Huang [1], Dominique A. Garrison [1], Zahra Talebi [1], Alice A. Gibson [1], Yan Jin [1], Mahesh Nepal [1], Ingrid M. Bonilla [2], Qiang Fu [1], Xinxin Sun [1], Alec Millar [3], Mikhail Tarasov [3], Christopher E. Jay [4], Xiaoming Cui [5], Heidi J. Einolf [5], Ryan M. Pelis [5], Sakima A. Smith [6,7], Przemysław B. Radwański [3,7], Douglas H. Sweet [4], Jörg König [8], Martin F. Fromm [8], Cynthia A. Carnes [3,7,9], Shuiying Hu [3] and Alex Sparreboom [1,*]

[1] Division of Pharmaceutics and Pharmacology, College of Pharmacy and Comprehensive Cancer Center, The Ohio State University, Columbus, OH 43210, USA; uddin.33@osu.edu (M.E.U.); eisenmann.11@buckeyemail.osu.edu (E.D.E.); li.10991@osu.edu (Y.L.); huang.2834@buckeyemail.osu.edu (K.M.H.); garrison.220@buckeyemail.osu.edu (D.A.G.); talebi.9@osu.edu (Z.T.); gibson.972@osu.edu (A.A.G.); jin.1134@osu.edu (Y.J.); nepal.17@osu.edu (M.N.); pkpdfu@gmail.com (Q.F.); xinxin224@gmail.com (X.S.)

[2] Department of Physiology and Cell Biology, College of Medicine, The Ohio State University, Columbus, OH 43210, USA; ingrid.bonilla4@upr.edu

[3] Division of Outcomes and Translational Sciences, College of Pharmacy, The Ohio State University, Columbus, OH 43210, USA; alec.miller@midwestern.edu (A.M.); tarasov.4@osu.edu (M.T.); radwanski.2@osu.edu (P.B.R.); carnes.4@osu.edu (C.A.C.); hu.1333@osu.edu (S.H.)

[4] Department of Pharmaceutics, School of Pharmacy, Virginia Commonwealth University, Richmond, VA 23298, USA; jayce@mymail.vcu.edu (C.E.J.); dsweet@vcu.edu (D.H.S.)

[5] Novartis Institute for Biomedical Research, East Hanover, NJ 07936, USA; xiaoming.cui@novartis.com (X.C.); heidi.einolf@novartis.com (H.J.E.); ryan.pelis@novartis.com (R.M.P.)

[6] OSU Wexner Medical Center, Department of Internal Medicine, Division of Cardiovascular Medicine, The Ohio State University, Columbus, OH 43210, USA; sakima.smith@osumc.edu

[7] Dorothy M. Davis Heart and Lung Research Institute, The Ohio State University, Columbus, OH 43210, USA

[8] Institute of Experimental and Clinical Pharmacology and Toxicology, Friedrich-Alexander-Universität Erlangen-Nürnberg, 91054 Erlangen, Germany; joerg.koenig@fau.de (J.K.); martin.fromm@fau.de (M.F.F.)

[9] Division of Pharmacy Practice and Science, College of Pharmacy, The Ohio State University, Columbus, OH 43210, USA

* Correspondence: sparreboom.1@osu.edu

This PDF file includes

Tables S1 to S5
Figures S1 to S4

Table S1. Pharmacokinetic parameters of dofetilide after i.v. administration (2.5 mg/kg) in mice.

Group	C_{max} (ng/ml)	C_{max} (Fold-change)	AUC_{last} (ng×h/ml)	AUC_{last} (Fold-change)
**Female**				
Wild-type	1640 ± 170		673 ± 28.0	
OCT1/2$^{-/-}$	1960 ± 126	1.29	756 ± 47.0	1.19
MATE1$^{-/-}$	1520 ± 149	0.93	635 ± 61.0	0.94
**Male**				
Wild-type	1540 ± 131		587 ± 29.4	
OCT1/2$^{-/-}$	1120 ± 113	0.81	461 ± 23.0	0.76
MATE1$^{-/-}$	1380 ± 112	0.90	603 ± 28.5	1.03

Data represent mean ± SEM.

Abbreviations: C_{max}, peak plasma concentration; AUC_{last}, area under the plasma concentration-time curve to the last measurable concentration.

Table S2. Influence of contraindicated drugs on the pharmacokinetic parameters of dofetilide (5 mg/kg) in female mice.

Concurrent treatment	C_{max} (ng/ml)	C_{max} (Fold-change)	AUC_{last} (ng×h/ml)	AUC_{last} (Fold-change)
**None**				
Wild-type	191 ± 13.0		329 ± 24.5	
OCT1/2$^{-/-}$	218 ± 18.0	1.14	362 ± 25.0	1.10
MATE1$^{-/-}$	276 ± 45.4	1.44	346 ± 37.0	1.05
**Bictegravir (30 mg/kg)**				
Wild-type	365 ± 72.0*	1.91	595 ± 194*	1.81
OCT1/2$^{-/-}$	282 ± 132	1.47	354 ± 46.4	1.08
MATE1$^{-/-}$	383 ± 86.4*	2.00	614 ± 77.0*	1.87
**Cimetidine (100 mg/kg)**				
Wild-type	656 ± 81.3***	3.43	1400 ± 183***	4.25
OCT1/2$^{-/-}$	420 ± 89.4**	2.20	1270 ± 150***	3.86
MATE1$^{-/-}$	625 ± 92.2***	3.27	1400 ± 89.5***	4.27
**Ketoconazole (50 mg/kg)**				
Wild-type	315 ± 34.0*	1.64	588 ± 64.0**	1.79
OCT1/2$^{-/-}$	475 ± 61.5**	2.48	1090 ± 128***	3.31
MATE1$^{-/-}$	455 ± 32.0***	2.38	938 ± 181***	2.85
**Trimethoprim (100 mg/kg)**				
Wild-type	264 ± 27.0	1.38	428 ± 25.4	1.30
OCT1/2$^{-/-}$	220 ± 38.0	1.15	440 ± 76.0	1.34
MATE1$^{-/-}$	212 ± 45.0	1.11	345 ± 29.3	1.05
**Verapamil (10 mg/kg)**				
Wild-type + Verapamil	518 ± 47.0***	2.71	602.0 ± 35.0*	1.83
OCT1/2$^{-/-}$ + Verapamil	519 ± 42.4***	2.71	669.0 ± 42.0**	2.04
MATE1$^{-/-}$ + Verapamil	384 ± 58.0**	2.01	638.0 ± 26.5**	1.94

Data represent mean ± SEM. *P < 0.05, **P < 0.01, ***P < 0.001 versus single agent dofetilide.

Abbreviations: C_{max}, peak plasma concentration; AUC_{last}, area under the plasma concentration-time curve to the last measurable concentration.

Table S3. Influence of ketoconazole or verapamil on the pharmacokinetics of dofetilide (5 mg/kg) in male mice.

Concurrent treatment	C_{max} (ng/ml)	C_{max} (Fold-change)	AUC_{last} (ng×h/ml)	AUC_{last} (Fold-change)
**None**				
Wild-type	151 ± 8.80		278 ± 12.3	
OCT1/2$^{-/-}$	191 ± 23.4	1.26	351 ± 36.0	1.26
MATE1$^{-/-}$	235 ± 50.0*	1.55	407 ± 32.6**	1.46
**DDI with Ketoconazole (50 mg/kg)**				
Wild-type	415 ± 43.0***	2.75	728 ± 55.4***	2.62
OCT1/2$^{-/-}$	453 ± 65.0***	3.00	1050 ± 142***	3.77
MATE1$^{-/-}$	509 ± 62.2***	3.36	894 ± 92.0***	3.21
**DDI with Verapamil (10 mg/kg)**				
Wild-type	181 ± 32.0	1.20	259 ± 18.3	0.93
OCT1/2$^{-/-}$	148 ± 32.0	0.98	328 ± 52.0	1.18
MATE1$^{-/-}$	246 ± 38.4	1.63	406 ± 38.0	1.46

Data represent mean ± SEM. *P < 0.05, **P < 0.01, ***P < 0.001 vs single agent dofetilide.

Abbreviations: C_{max}, peak plasma concentration; AUC_{last}, area under the plasma concentration-time curve to the last measurable concentration.

Table S4. Observed and predicted pharmacokinetic parameters of dofetilide with the presence and absence of cimetidine and ketoconazole.

	Mean C_{max} ± SD (CV%) (ng/mL)		Mean AUC_{0-last} ± SD (CV%) (ng×h/ml)		Mean CL ± SD (CV%) (L/h)	
	**Predicted**	**Observed**	**Predicted**	**Observed**	**Predicted**	**Observed**
Dofetilide (0.5 mg BID, p.o.)	2.48 ± 0.58 (23)<br>PE = +32.66%	1.87 ± 0.65	18.0 ± 3.38 (19)<br>PE = +2.91%	17.5 ± 3.2	28.8 ± 6.05 (21)<br>PE = +30.8%	22.0
Dofetilide (0.5 mg i.v. infusion over 90 min)	4.28 ± 0.52 (12)<br>PE = +38.06%	3.1 ± 0.50	20.4 ± 3.92 (19)<br>PE = -3.92%	21.2 ± 4.5	25.4 ± 4.94 (19)<br>PE = +40.48%	18.1
**DDI with Cimetidine 400 mg BID (p.o.)**						
Dofetilide (0.5 mg, p.o.)	2.84 ± 0.53 (19)<br>PE = +25.7%	2.26	19.6 ± 3.66 (19)<br>PE = +12.6%	17.4	26.4 ± 5.36 (20)<br>PE = +28.2%	20.6
Dofetilide + Cimetidine	3.98 ± 0.70 (18)<br>PE = +15.7%	3.44	32.4 ± 6.76 (21)<br>PE = +17.9%	27.5	16.1 ± 3.79 (23)<br>PE = +39.1%	11.6
% Change	40.1% ↑	52.2% ↑	65.5% ↑	58.0% ↑	38.9% ↓	43.7% ↓
**DDI with Ketoconazole 400 mg QD (p.o.)**						
Dofetilide (0.5 mg, p.o.)	2.87 ± 0.52 (18)<br>PE = +27.0%	2.26	19.9 ± 3.69 (19)<br>PE = +14.3%	17.4	26.0 ± 5.20 (20)<br>PE = +26.3%	20.6
Dofetilide + Ketoconazole	3.70 ± 0.73 (20)	-	28.1 ± 6.17 (22)	-	18.7 ± 4.68 (25)	-
% Change	28.9% ↑	53.0% ↑	41.1% ↑	41.0% ↑	28.0%↓	-

Abbreviations: p.o., per oral; BID, twice per day; QD, once a day, C_{max}, peak plasma concentration; AUC_{0-last}, area under the plasma concentration-time curve between time zero and the last measurable concentration; CL, clearance; PE, calculated prediction error (%) = [(predicted value - observed value)/observed value] × 100.

Table S5. Input parameters of dofetilide in PBPK model.

Input parameters	Description	Units	Value	Reference
**1. Physicochemical and binding properties**				
MW	Molecular weight	g/mol	441.57	
Log P	Octanol-water partition	-	2.1	PubChem
Compound type	Acid,base or neutral	-	Monoprotic base	
pK_a	Acid dissociation constant	-	7.89	UWDIDB
B/P profile	Blood to plasma ratio dependent on drug concentration	-	1.048	Simcyp predicted
fu	Fraction unbound in plasma	-	0.36	UWDIDB
**2. Absorption**				
Absorption model	First order absorption model			
f_a	Fraction available from dosage form	-	1	
CV f_a	Coefficient of variation fa	%	30	Default
k_a	Absorption rate constant	1/h	0.9	UWDIDB
CV k_a	Coefficient of variation ka	%	30	Default
fu_{gut}	Unbound fraction in enterocytes	-	0.039	Simcyp predicted
Q_{gut}	Nominal flow in gut model	L/h	4.068	Simcyp predicted
CV Q_{gut}	Coefficient of variation Q(gut)	%	30	Default
$P_{eff,man}$	Effective permeability in man	10^{-4} cm/s	12	Simcyp predicted
Permeability assay	Passive + active permeability		Physicochemical	
PSA	Polar surface area	Å^2	104.81	Drug central
HBD	Hydrogen bond donor		2	Drug central
**3. Distribution**				
Distribution model	Full PBPK model			
Prediction method			Method 2	
Kp scalar	Tissue to plasma partition coefficient		0.61	Optimized

Input parameters	Description	Units	Value	Reference
V_{ss}	Volume of distribution at steady-state	L/kg	2.8	Smith et al 1992 [16]
CV V_{ss}	Coefficient of variation V_{ss}	%	30	Default
**4. Elimination**				
Elimination model	Enzyme kinetics			
CYP, recombinant			CYP3A4	Smith et al 1992 [16]
CL_{int} (HLM)	*In vitro* clearance (human liver microsomes)	μL/min/mg protein	0.25	Smith et al 1992 [16]
fu_{mic}	Fraction unbound *in vitro*		0.81	
CLr	Renal clearance in 20-30yr healthy male	L/h	18.06	Smith et al 1992 [16]
**5. Transport**				
Organ/Tissue			Kidney	
Transporter			*SLC22A2* (OCT2)	
Location			Basolateral	
Function			EGD model	
J_{max}		pmol/min/10^6 cells	506.35	Measured
K_m	Michaelis-Menten constant	μM	347.2	Measured
System			User	
RAF/REF	Relative activity factor/relative expression factor		1	Optimized
CL_{PD} basal	Passive diffusion clearance	mL/min/10^6 cells	0	
Transporter			*SLC47A1* (MATE1)	
Location			Apical	
Function			Efflux	
CL_{int}	Intrinsic clearance	μL/min/10^6 cells	22.2	Measured
System			User	
RAF/REF	Relative activity factor/relative expression factor		0.25	Optimized

Input parameters	Description	Units	Value	Reference
CL_{PD} apical	Passive diffusion clearance	mL/min/10^6 cells	0	

Drug central, https://drugcentral.org/drugcard/942

UWDIDB, University of Washington Drug Interaction Database

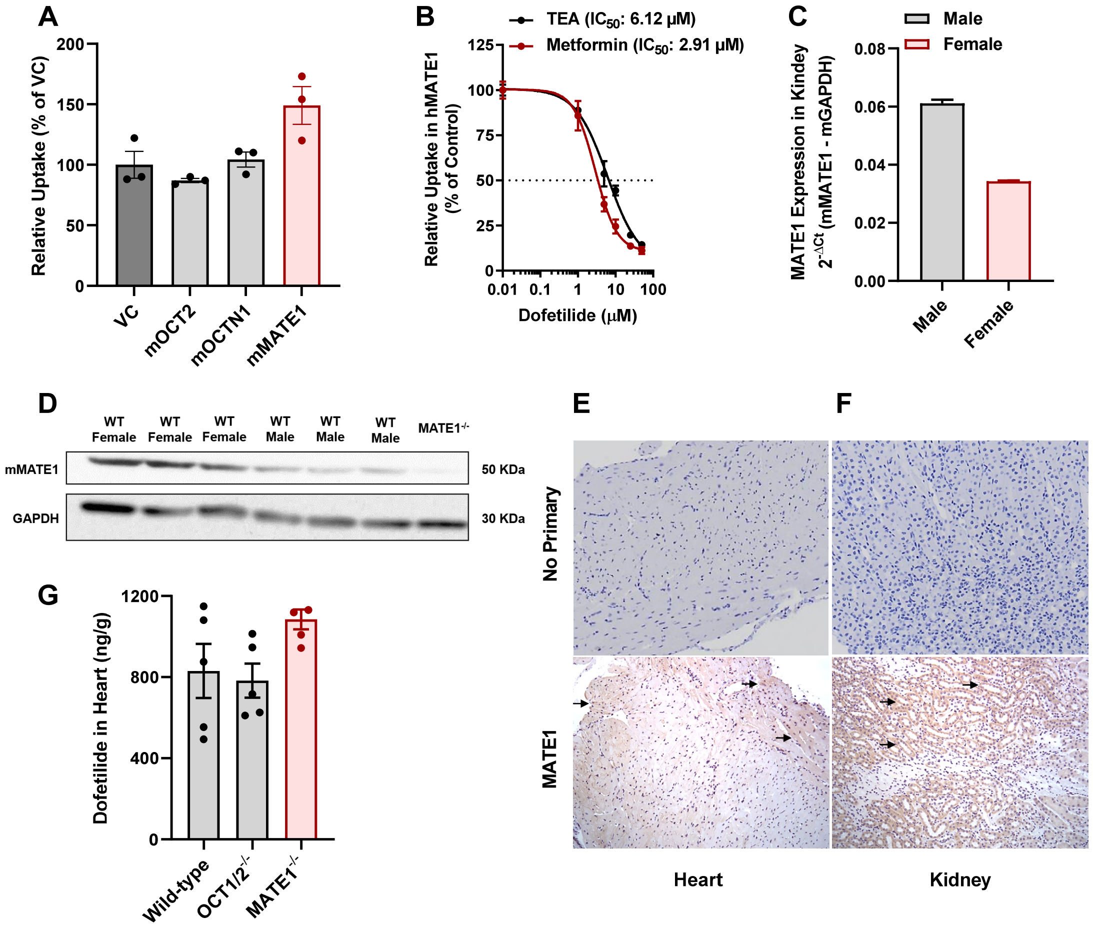

Figure S1. **Influence of MATE1 on the transport of dofetilide. (A)** Transport of [^{3}H] dofetilide (1 μM) in cells overexpressing mouse (m) OCT2, OCTN1, or MATE1. Relative uptake is expressed as percentage change compared with empty vector controls (n = 3). **(B)** Uptake of [^{14}C] TEA (2 μM) and [^{14}C] metformin (5 μM) in HEK293 cells overexpressing human (h) MATE1 after preincubation with dofetilide at various concentrations (1-50 μM) for 15 min, followed by the co-incubation with TEA and metformin for 15 min. Data represent the mean ± SEM and are expressed as a percentage over vector control. **(C)** Gene expression of MATE1 in the kidney isolated from untreated male and female wild-type mice (n = 4 per group). **(D)** Protein expression of MATE1 in the heart isolated from untreated wild-type (WT) and MATE1$^{-/-}$ mice (n=3). Immunohistochemical detection of MATE1 (bottom panels) in the wild-type mouse heart **(E)** and kidney **(F)**. Tissue staining lacking the primary antibody was performed as a negative control for each tissue group (top panels). Representative images were taken at x40 magnification. **(G)** Concentration of dofetilide in whole heart tissue from male wild-type, OCT1/2-deficient, and MATE1-deficient mice 15 min after a single i.v. injection of dofetilide at a dose of 2.5 mg/kg (n = 4-5 per group).

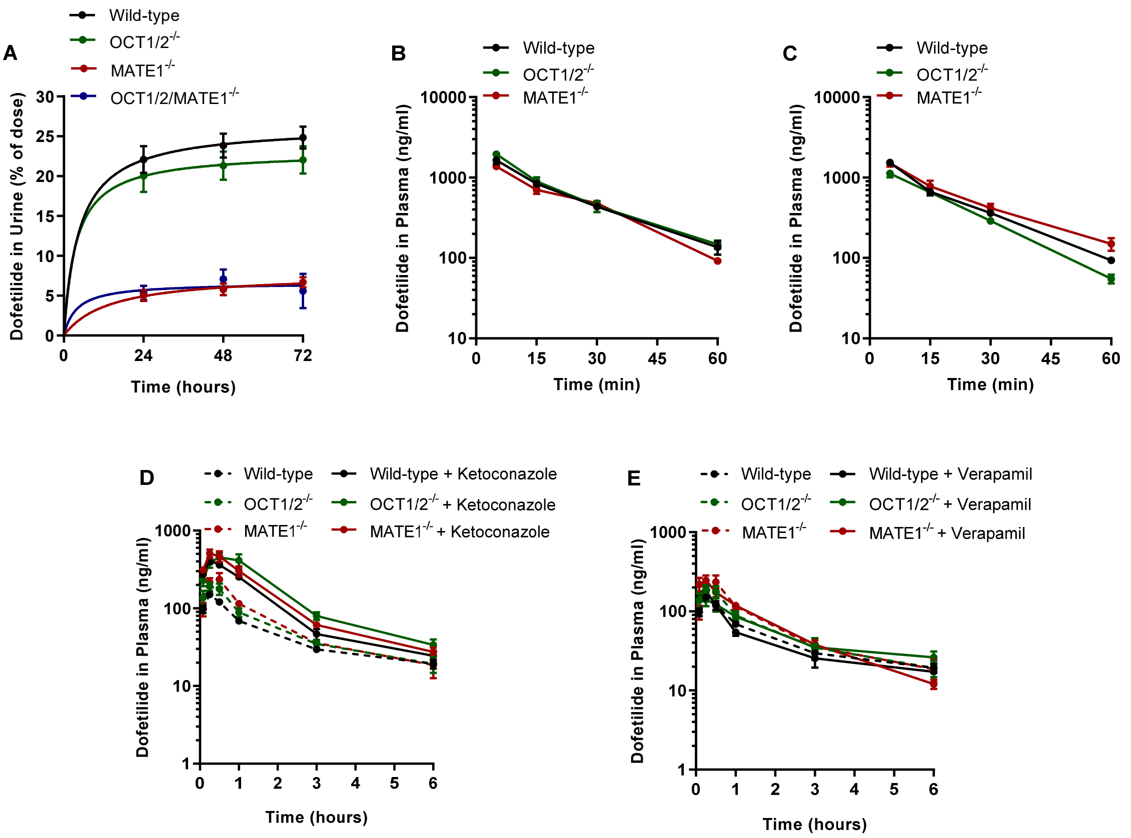

Figure S2. Influence of mouse genotype and ketoconazole or verapamil treatment on the pharmacokinetics of dofetilide. (A) Urinary excretion of dofetilide in male wild-type, OCT1/2-deficient, MATE1-deficient, and OCT1/2/MATE1-deficient mice (n = 5) following an i.v. dose of dofetilide (2.5 mg/kg). Plasma concentration time profiles of dofetilide in female **(B)** and male **(C)** wild-type, OCT1/2-deficient, and MATE1-deficient mice (n = 5 per group) receiving a single i.v. dose of dofetilide (2.5 mg/kg). Plasma concentration time profiles of dofetilide in wild-type, OCT1/2-deficient, and MATE1-deficient male mice (n = 5 to 38 per group) receiving an oral dose of dofetilide (5 mg/kg) 30 min after the administration of ketoconazole (50 mg/kg) **(D)** or verapamil (10 mg/kg) **(E)**.

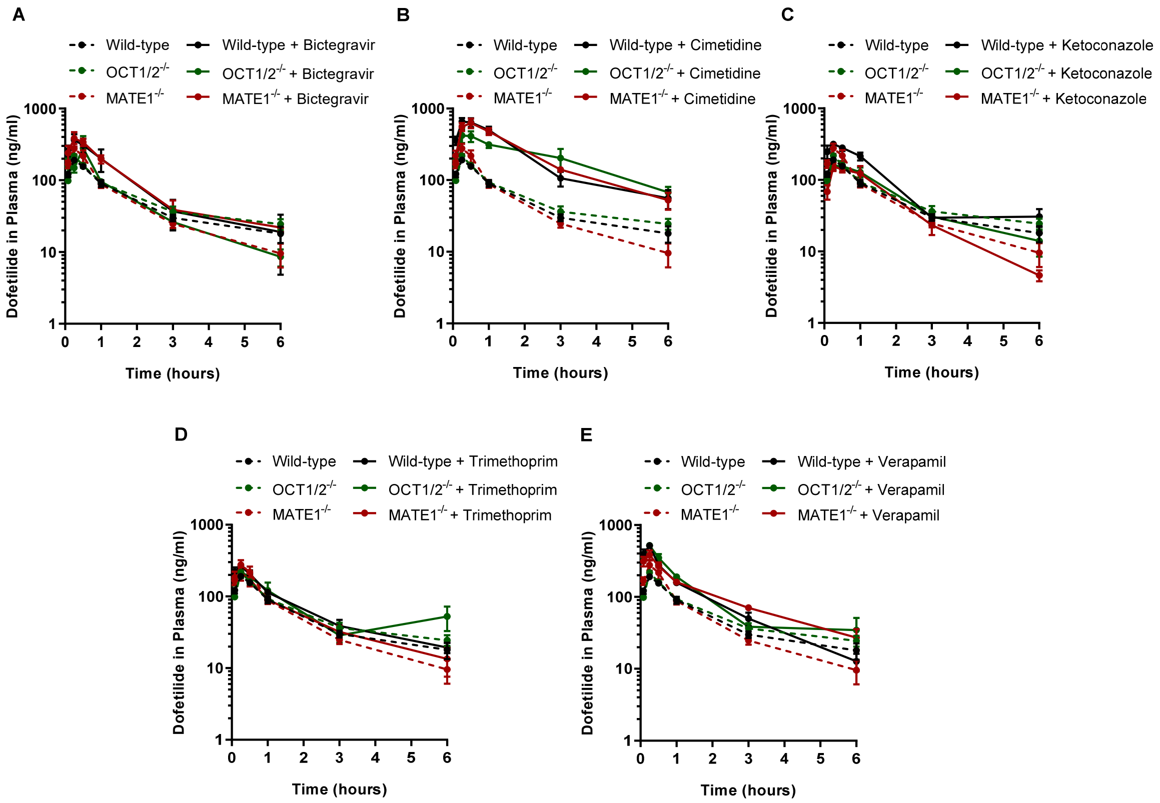

Figure S3. Drug-drug interactions between dofetilide and contraindicated drugs. Plasma concentration time profiles of dofetilide in female wild-type, OCT1/2-deficient, and MATE1-deficient mice (n = 5 to 29 per group) receiving a single oral dose of dofetilide (5 mg/kg) 30 min after the administration of bictegravir (30 mg/kg) **(A)**, cimetidine (100 mg/kg) **(B)**, ketoconazole (50 mg/kg) **(C)**, trimethoprim (100 mg/kg) **(D)**, or verapamil (10 mg/kg) **(E)**.

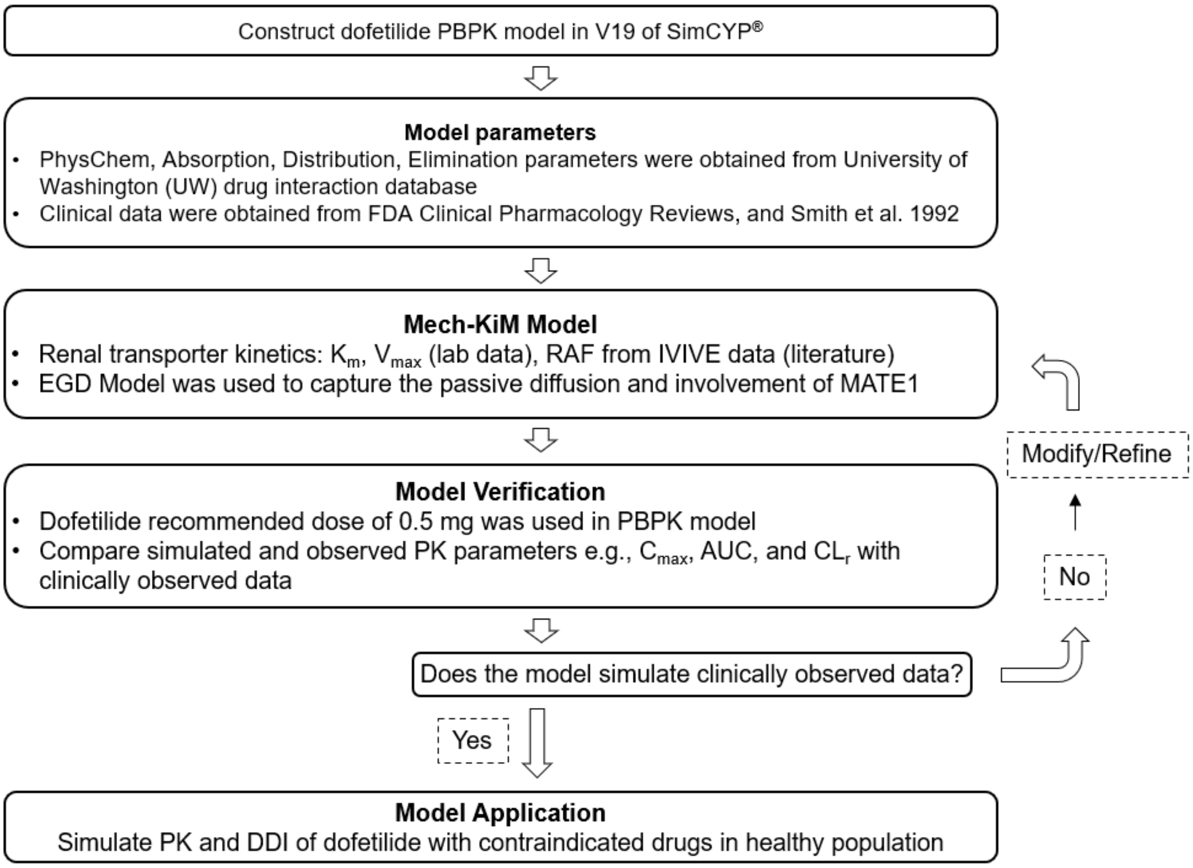

Figure S4. Workflow of the development of physiologically based pharmacokinetic (PBPK) model.

Abbreviations: AUC, area under the curve; C_{max}, peak plasma concentration; CL, clearance; FDA, U.S. Food and Drug Administration; IVIVE, *in vitro* to *in vivo* extrapolation; K_m, Michaelis-Menten constant; RAF, relative activity factor; PK, pharmacokinetics; V_{max}, maximum velocity.
